# The use of airborne laser scanning to develop a pixel-based stratification for a verified carbon offset project

**DOI:** 10.1186/1750-0680-6-9

**Published:** 2011-10-17

**Authors:** Jordan Golinkoff, Mark Hanus, Jennifer Carah

**Affiliations:** 1 The Conservation Fund, 14951 "A" Caspar Road, Box 50, Caspar, CA 95420, USA; 2 GeoDigital International, McMaster Innovation Park, 175 Longwood Road South, Suite 400A, Hamilton, ON L8P 0A1, Canada; 3 The Nature Conservancy, California Regional Office, 201 Mission St., 4th Floor, San Francisco, CA 94105, USA

**Keywords:** Forest carbon offsets, MRV, LiDAR, Airborne Laser Scanning, stratification, post-stratification, carbon project, carbon stock estimation

## Abstract

**Background:**

The voluntary carbon market is a new and growing market that is increasingly important to consider in managing forestland. Monitoring, reporting, and verifying carbon stocks and fluxes at a project level is the single largest direct cost of a forest carbon offset project. There are now many methods for estimating forest stocks with high accuracy that use both Airborne Laser Scanning (ALS) and high-resolution optical remote sensing data. However, many of these methods are not appropriate for use under existing carbon offset standards and most have not been field tested.

**Results:**

This paper presents a pixel-based forest stratification method that uses both ALS and optical remote sensing data to optimally partition the variability across an ~10,000 ha forest ownership in Mendocino County, CA, USA. This new stratification approach improved the accuracy of the forest inventory, reduced the cost of field-based inventory, and provides a powerful tool for future management planning. This approach also details a method of determining the optimum pixel size to best partition a forest.

**Conclusions:**

The use of ALS and optical remote sensing data can help reduce the cost of field inventory and can help to locate areas that need the most intensive inventory effort. This pixel-based stratification method may provide a cost-effective approach to reducing inventory costs over larger areas when the remote sensing data acquisition costs can be kept low on a per acre basis.

## Background

The world's forests are a critical sink of carbon dioxide [1]. It is estimated that forest degradation or destruction results in 6 to 17% of total anthropogenic CO_2 _emissions annually [2]. Because of the importance of forest ecosystems in adapting to and mitigating climate change, there are now many policy initiatives to preserve and restore forest ecosystems for a climate benefit [3,4]. Despite years of discussion however, policies to reduce emissions from terrestrial ecosystems have generally not been adopted. An exception to this is California's cap and trade system that will incorporate carbon offsets starting in 2012 (barring a legal challenge) - see [5].

In part due to the dearth of climate change policies, a vibrant voluntary carbon offset market has sprung up centered around a suite of different carbon project standards [6-9], and managing forests for carbon offsets can provide an important income stream for landowners willing to undertake the costs and requirements of these standards. These standards all have slightly different requirements regarding how to quantify the amount of carbon offsets generated, but generally all require periodic ground-based installation and measurement of plots to monitor project level carbon storage. This paper will focus on the requirements of the Climate Action Reserve Forest Project Protocol as this protocol is substantially similar to what will likely be adopted by the state of California for their compliance carbon market system. The ground based inventory described here, like most traditional forest monitoring, relies on tree measurement and conversion to volume, biomass, and carbon equivalents using established species-specific regressions developed through destructive sampling of trees [10-13]. These sample-based estimates of forest carbon storage are then extrapolated across the full project, often through a stratification approach, whereby unsampled areas receive estimates from areas with similar characteristics based on their remotely sensed attributes [14].

This traditional approach to estimating forest parameters has recently been supplemented and improved upon with the use of remote sensing technologies like Light Detection and Ranging data (LiDAR) paired with high resolution multi-spectral imagery. While these new technologies can accurately estimate forest carbon stocks and fluxes, some of the methods are not easily applicable to forest carbon offset projects because of their complexity and expense. There is a need to apply these new remote sensing products in the context of the voluntary carbon market to show their usefulness at a project level in conformance with typical forest carbon project standards.

### ALS and Optical Remote Sensing

Optical remote sensing products derived from airborne and satellite-borne sensors - Landsat Thematic Mapping Imagery [15,16], IKONOS imagery [17], Quickbird imagery [17-20], SPOT HRG imagery [21], Moderate Resolution Imaging Spectroradiometer (MODIS) [22-28], and others [29,30]- have all been used to classify forest landscapes and in some cases to estimate standing carbon stocks. However, estimates of carbon stocks and classifications created using optical sensors alone usually have trouble differentiating areas with high carbon stocks [31,32]. Synthetic Aperture Radar (SAR) sensors can help improve estimates of biomass but these sensors also saturate in high biomass systems [33]. Because of these limitations, the estimation of forest carbon stocks is often greatly improved with data about forest structure and specifically forest height. Airborne Laser Scanning (ALS), provides a richer summary of forest conditions and more accurate estimates of volume and biomass due to its ability to accurately capture forest heights (LiDAR intensity values can also be used to improve estimates).

ALS paired with other optical remote sensing data is a well-established approach to spatially estimating forest attributes [34-40]. The use of optical remote sensing data in conjunction with LiDAR data is helpful in both delineating crown boundaries and in differentiating between species [32,35,37-40]. The ability to make species level distinctions is especially important when estimating merchantable timber volumes and biomass, as these parameters differ between species in trees that are the same size.

ALS data is collected from an instrument that is flown over the forest on an airplane or helicopter. Laser pulses emitted from an airborne instrument reflect off of terrain and vegetation revealing both forest structure (e.g. - height, sub-canopy elements) and a detailed digital elevation model [41,42]. Individual laser returns can be discrete or continuous (waveform). The spatial resolution can vary from many returns per square meter to sparser returns. The coverage of the ALS can range between full coverage of a given area with no gaps to a sample of the area based on transects below the flight lines to spot samples within transects (i.e. GLAS) [43,44].

There are two broad categories of ALS data analysis approaches: area based approaches (ABA)/statistical canopy height distribution approaches, and individual tree crown approaches (ITC). Many individual tree approaches use the cloud of LiDAR point data and their relationship to neighbourhood points to build individual crown polygons and/or 3-dimensional tree profiles [42,45,46]. These individual tree records can then be aggregated to any scale required to create stand level estimates. These ITC approaches use both parametric and non-parametric approaches [47].

In area based approaches, plot level data is related to remote sensing data that has been aggregated to pixel, plot, or polygon (e.g. stand) units to estimate volume, biomass, or other area based metrics. Area based approaches fall broadly into two main categories:

1) The first category relates grid-cell or stand level remote sensing data to measured plot characteristics to build parametric models to represent forest data. These models have been shown to explain the vast majority of the variation in tree height, diameter at breast height, volume, biomass, basal area, and a suite of other parameters [36-38,41,48-51]

2) The second broad category uses non-parametric classification or nearest neighbour methods to stratify the forest into similar groups [52-58]. Non-parametric approaches include k-nearest neighbour techniques [59] and classification algorithms such as Random Forests [53].

Area-based approaches and individual tree approaches to estimating forest parameters are not mutually exclusive however, and several authors have shown how area based systems can be combined with individual tree methods [40,60].

### ALS and Optical Remote Sensing for a Forest Carbon Offset Project

The methods outlined above all provide different approaches to using ALS data and other data sources to estimate forest parameters. There are two main hurdles in using these methods for forest carbon offset projects. First, the method must be cost-effective and must also fit within the existing management framework of the project. Second, the estimation method must meet the monitoring and verification requirements of the carbon offset protocol. These protocols require periodic inventory of the forest and the application of species-level biomass and carbon conversion equations to all inventory estimates [7,8,61]. For example, the Climate Action Reserve Forest Project Protocol v3.2 requires that the United States Forest Service biomass conversions are used for all trees in the project area. Using a stratified inventory approach provides an easily understandable way to generate strata-level tree lists simply from plot data and because of this is more easily verified [8]. Although it may be possible to use some of the existing approaches within a forest carbon project framework, their complexity makes them difficult to understand and potentially challenging to verify. Some approaches do not generate species specific estimates of tree size that can then be used to expand to volume and/or biomass using approved biomass regressions (e.g. - [36]). **The primary objective of this paper will be to describe how the ALS and optical remote sensing stratification system adequately meets the requirements of forest carbon protocols while improving the accuracy of forest inventory estimates**.

In addition to describing a method for ALS and optical remote sensing data to stratify a forest ownership to meet the requirements of a carbon project protocol, this paper will also detail how and where sampling should occur. ALS and optical remote sensing data provide a wealth of information that can be used to increase the efficiency of sampling a forest. **A secondary objective of this paper then, is to provide a method to choose the optimal size for the units of analysis (grid-cell size) and to locate plots across the project once the grid is established**. Past research has used LiDAR data to stratify an area and locate field plots but these studies have not combined both LiDAR and optical data in the stratification and plot location. These studies have shown that using LiDAR data to first stratify an area and then to locate field plots based on initial strata reduced the root mean squared error (RMSE) of predicted volume [44,62].

The question of the optimal grid-cell size has been addressed from the opposite direction by Gobakken and Næsset [63]. They examined the optimum plot size to use to best correlate the remote sensing data with the inventory data; however their analysis only used fixed area plot designs and did not examine what scale to aggregate the remote sensing data (i.e. - how big should the grid cells be?). Van Aardt et al. [64] examined various sizes of stands using variable radius plots but their analysis involved the best fit when a stand could contain multiple plots and did not use a regular grid system. Therefore, this new approach will show how to find the most appropriate grid cell size that relates variable radius prism plots to remotely sensed data where each grid cell receives no more than one plot.

Although there has been ample discussion of the technical nature of ALS-assisted forest estimation, few studies move beyond the initial analysis and results with an eye to future management and monitoring. **The third and final objective of this study is to examine how to best leverage data generated by this stratification and modelling exercise for typical management purposes and how to perform inventory updates assuming regular remote sensing data acquisition is not feasible (given cost constraints)**.

**Figure 1:**
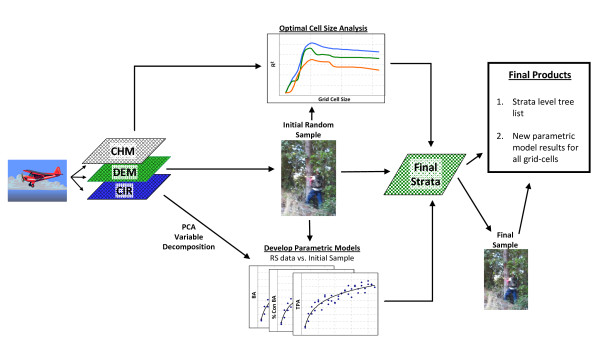
**Outline of ALS and optical remote sensing data stratification method**.

Using an ALS and optical remote sensing stratification system, a verified and registered carbon project in Mendocino County, California, the Garcia River Forest (GRF), was inventoried in 2010 to meet the requirements of the California Climate Action Reserve (CAR) Forest Project Protocol. Three remotely sensed image datasets - color infrared data (CIR), Red, Green, and Blue true colour imagery (RGB), and LiDAR data - were used to create a canopy segment layer, a canopy height model, and a digital elevation model. These data were summarized to 20 m (1/10 acre) grid cells over the property. An initial systematic random sample was then installed over the full property. The remotely sensed variables were collapsed using a principal components analysis, and combined with the canopy segment summary variables and topographic descriptors, and field survey data to explain the variation in the initial sample of basal area (BA) using a regression model (models to predict trees per hectare (TPH) and percent conifer BA were also developed). The BA model was then used to estimate the basal area for each grid-cell on the property. The BA modelled estimates were then combined with average canopy height derived from the LiDAR canopy height model and the product of basal area and canopy height was calculated as a proxy of volume. This proxy was then divided into classes using an optimal binning heuristic, to define the strata. After this final stratification was completed, a second set of plots were installed to fully inventory each strata, with the number of plots based on the variability of each strata (see Figure 1).

## Results

### Traditional Stratification and Inventory and Approaches

Traditional forest stand delineation and stratification (typing) are done by examining aerial photos of a forest and manually drawing boundaries around similar forest areas. This approach requires a forester to then place each stand into a stratum, based on their familiarity with actual forest conditions. This stratification may also use a visual check of ground data and may incorporate some plot data to inform how stands are assigned to strata [65-68].

**Figure 2:**
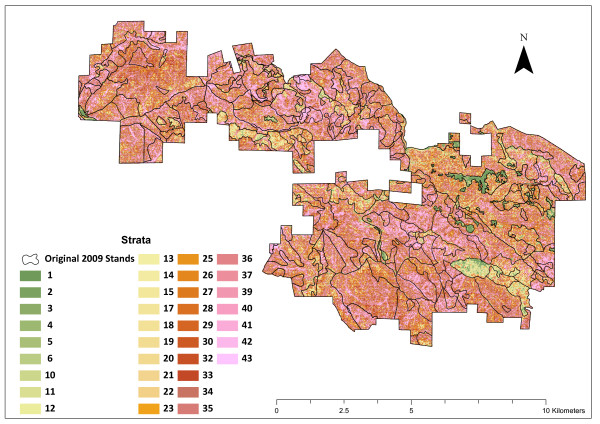
**Overlay of 2009 Stand Layer with final stratification of the Garcia River Forest**.

This approach to stand-delineation and stratification is preferred to unstratified sampling designs, both because of its simplicity and its accuracy in estimating forest parameters. This approach is also preferred because knowing stand boundaries is useful for management purposes and harvest planning. The use of forest strata and stand delineation is ideal in forests with well-documented management histories and/or areas where even-age management was used in the past. Stand boundaries are easily seen and delineated when they correspond to past management and management history can inform the typing of stands. However, in forests managed with uneven-aged silvicultural systems or without a well maintained history of past management, it can be difficult to create a stand map that accurately partitions the variability of a forest due to the relative homogeneity of the forest when observed from aerial photos. In this study, the field site fits within one of these categories: the past management was well-documented but the uneven-aged harvests have left a forest that does not have many clear stand boundaries (see Figure 2), thus rendering the traditional stratification approach less accurate.

Using an ALS and optical remote sensing stratification system, the 9,623 ha (23,780 acre) GRF property was divided into 36 strata (35 forested and 1 non-forested) across the property. Each strata is at least 4.05 ha (10 acres) in size. Strata with higher numbers generally represent better stocked forest areas that have larger trees with more volume and carbon. This approach to forest stratification produces inventory estimates with more statistical confidence relative to the traditionally stand-based inventory approach using about half as many plots (see Table 1 and Table 2). Figure 2 shows a map of the strata generated by this new approach with the old stand boundaries shown in black. Except for the green areas that correspond with grassland, brush-fields, true oak woodlands, or stands treated to reduce tanoak competition most of the property has unclear stand boundaries in a traditional sense, with a high degree of variability within stands.

**Table 1:** Inventory Accuracy Statistics

Sample Type	Original Forest Inventory: (Multi-Stage Probability Proportional To Size Stand Based Stratification)	ALS and ORS Grid-Based Inventory: (post-stratification)
**C 90% Accuracy**	3.72%	3.42%

**BA 90% Accuracy**	5.4%	3.60%

**BF 90% Accuracy**	7.56%	5.30%

The original forest accuracy estimates are based on all plots grown forward to 2009 using the Forest Projection and Planning System growth and yield model calibrated to the Northern California redwood region. The 90% accuracy percentage is the property level standard error of the mean multiplied by the 90% t-value (1.645) divided by the mean value.

**Table 2:** Summary and Comparison of 2009 and 2010 Stratification Systems

	2009	2010
**Total Plots**	1579	810

**Max Plots/Strata**	394	40

**Min Plots/Strata**	4	15

**Median Plots/Strata**	45	22

**Average Plots/Strata**	75	23

**Total Stands (Pixels)**	278	240,410

**Sampled Stands (Pixels)**	170	810

**Max Stand (Pixel) Area (ha)**	1,023	0.04

**Min Stand (Pixel) Area (ha)**	0.8	0.04

**Median Stand (Pixel) Area (ha)**	14	0.04

**Mean Stand (Pixel) Area (ha)**	33	0.04

**Forested Strata #**	21	35

**Max Strata (ha)**	1,704	1,816

**Min Strata (ha)**	7.3	3.9

**Median Strata (ha)**	230	76

**Average Strata (ha)**	444	255

The 2010 "stands" are called stands as that is their closest analogue when thinking about a traditional stand-based stratified forest inventory. However, these "stands" do not correspond to management units and are therefore better thought of as pixels.

### Regression Model Results from the Initial 199 Plots

The model form used to explain the correlation in BA is shown below. Both the response and predictor variables have been transformed using a natural logarithm transformation.

Y=Xβ+ε

where Y is the transformed response, X is a matrix of transformed predictors identified by the Lasso method and β is the vector of least squares coefficients. The predictor variables used in these regressions are several topographic and LiDAR tree crown variables and the principle components of the color-infrared (CIR) and RGB imagery data sets as well as the PCA rotations for a suite of variables derived from the LiDAR data (the PCA rotations were used to reduce the number of parameters to analyze when building these regressions - see the Appendix for a full list of the predictor variables considered). The components of the β vector and the predictor variables (X) for the BA model are listed in Table 3. The variables are arranged such that those explaining most of the variation are listed first and those explaining the least are last. Regression relationships for trees per hectare and percent conifer BA are also shown below. These relationships were used when lumping strata with less than 10 acres into other larger strata in the last step of the stratification process. A logistic model form was used for % Conifer BA.

**Table 3:** Final Model Forms and Coefficients

BA		TPA		% Conifer BA	
**Intercept**	3.079788313	**Intercept**	6.19851	**Intercept**	-0.04949619

**CIR3**	-0.11917071	**Crown closure**	0.0006754	**LI1**	0.161971603

**Average crown segment height**	0.00519755	**LI6**	-0.19544	**RGB4**	0.81924046

**Crown closure**	0.017182801	**LI7**	0.05154	**LI2**	0.09321113

**LI7**	0.07755464	**LI4**	0.02984	**LI6**	-0.19769152

		**CIR6**	-0.11007	**RGB5**	-0.50907623

		**LI2**	-0.20571	**LI7**	0.294606256

		**LI1**	0.18478	**RGB1**	0.824221728

				**RGB6**	-0.42129326

				**LI5**	-0.50907623

All coefficients are significantly different from 0 at the 95% confidence level.

As has been found in previous crown-based inventory projects, the LiDAR and CIR based variables predict the BA and TPH components best, while LiDAR and RGB variables are more help in predicting species composition [32,37-40]. The dominance of the color variables in predicting species composition is likely due to the realized species composition of the property being better represented by the image spatial domain than the image frequency domain. The spatial domain treats the image plane as a spatially related database and summarizes the pixel information in context to its neighbors. The frequency domain works on the Fourier transformation of the pixel information. In this case texture, characterized by both grain size and arrangement were more important in discerning species composition than were the absolute color values [69]. In other environments where the leaf color differs more profoundly, color has been more important than texture.

Since the coefficient of determination (R^2^) is the square of the correlation (ρ) between the predicted and observed values, a simple transformation of it provides a measure of the sample efficiency (Table 4). Sample efficiency is the ratio of the number of correlated plots to uncorrelated plots required to achieve the same level of precision [70]. For example, using values from the table 4 a sample correlated to BA only would require 40.6% of the plots to achieve the same level of significance compared to an uncorrelated sample. This analysis is complicated since the goal of this project is to predict forest structure, which is a composite of these values (and others). The sampling efficiency therefore cannot be directly derived from these values; they are provided strictly as an illustration. However, if forest structure can be reduced to a single metric and that metric related to the remotely sensed data it is likely that the sample reduction would be even more significant (future efforts will likely sample based on Board Foot volume or total volume as this is more related to forest structure). Board Foot (BF) volume is the merchantable volume of trees and only is calculated for merchantable trees (i.e. - conifer species). This value is important for management purposes as BF volume is the primary economic value of many forests.

**Table 4:** Initial Model Fit Statistics

Model	MSE	R^2^	Sample efficiency = 2(1-ρ)	Number of variables
BA	0.21687	0.635	40.6%	4

TPA	1.46939	0.568	49.3%	7

%ConBA	1.95837	0.493	59.1%	10

**Figure 3:**
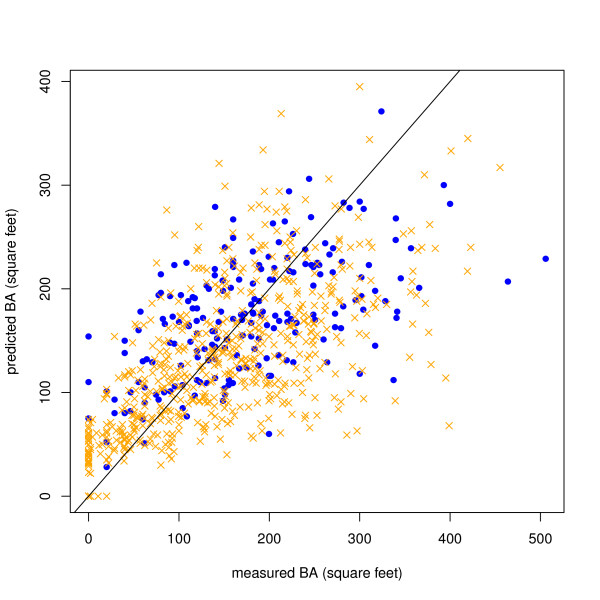
**BA Model residuals**. Initial sample: blue dots, final sample: orange x's. The BA model residuals were not significantly different than a normal distribution (Pearson Chi-Square Normality Test, p-value = 0.7076)

Figure 3 shows the modeled versus measured BA in the original and final plots. An examination of the model fit with the original 199 plots (blue) showed that there weren't any strong trends in the residuals.

### Final Stratification Results

The final ALS-optical remote sensing stratification system resulted in more accurate property level estimates of live and dead carbon and basal area than the prior traditional stratification system (Table 1). Accurate stand delineation has the goal of maximizing between-stand variance while minimizing within-stand variance. To better understand the improvement this new approach to stratification provides, it is compared to the previous inventory that used a traditional stand-based stratification.

Based on the results seen using this new stratification approach there are several conclusions that can be drawn. First, with half as many plots (Table 2), we have more statistical confidence in the inventory using this method due to the high resolution stratification derived from the remotely sensed imagery (Table 1). Second, this new stratification approach has shown that past samples most likely averaged more highly stocked riparian areas with non-riparian areas and therefore showed less volume on this property. Third, this new strata system allows for a flexible approach that can be easily leveraged when designing timber harvest plans or trying to understand the habitat of a given area. For example, accurate inventory estimates can now be made for any polygon across the full ownership simply by aggregating a set of grid cells.

### Old Stand Level Comparison

**Figure 4:**
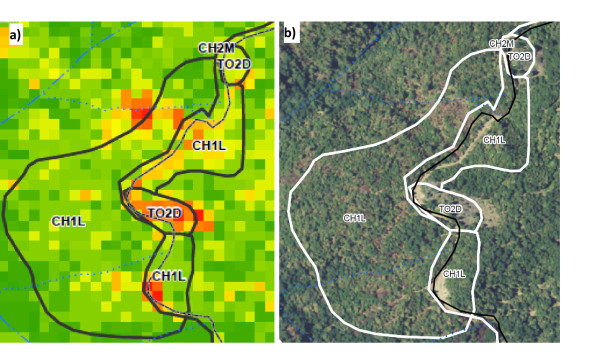
**A visual comparison of the current stratification system versus the prior system**. Figure 4a shows the current strataification system (with the old stand boundaries as well). The 0.04 ha grid cells are shaded to represent their different strata with redder cells having less volume than green cells. Figure 4b shows the prior stand delineation for this same area with the true-color imagery of the area as the base layer to show actual forest conditions. Note that the new strata grid-cells do correspond to the old stratification in areas where there are clear stand boundaries but in mixed forest conditions the new system can distinguish different forest conditions that the original strata system lumped together. This new strata system also does a much better job of mapping landings/clearings and wide road areas (most of the red and orange cells).

Visually, the strata systems are much different (see Figure 2 and Figure 4), as the old stand boundaries lump together many cells that are currently considered different strata. This visual comparison shows that although the old stratification and stand delineation does a reasonable job of capturing some of the differences in the stands, there are many areas where it is hard to see well defined stand boundaries.

Another way to compare the current strata system to the prior system is to look at some well sampled stands in the prior inventory and compare those estimates to the current strata-based estimates (Table 5). Quantitatively the differences between estimates of stand parameters are not statistically significant (except for BA - this result was also found in Hudak et al. [52] and they postulate that this bias is a result of the natural logarithm transformations and back transformations). These results therefore are an indication that the current stratification system, though much different than the previous system, produces estimates of stand level parameters that are similar to a traditional forest inventory (but more accurate). The advantage is that these estimates can now be found for any arbitrary polygon across the forest by grouping cells of interest and generating estimates for this group [52]. This approach therefore presents a much more flexible set of data to gauge forest conditions.

**Table 5:** Comparison of recently cruised stands using old strata system and current strata system

2009 Data (2008 Plot Data Is Grown to 2009)									2010 Data				
**Strata**	**Stand**	**Ha**	**Year Cruised**	**Plots**	**BA (m**^**2**^**/ha)**	**TPH (> 5 cm)**	**BF per ha**	**C (Mg/ha - no dead)**	**# of 2010 Strata**	**BA (m**^**2**^**/ha)**	**TPH (> 5 cm)**	**BF per ha**	**C (Mg/ha - no dead)**

**DR1M**	**2**	53	2009	4	47.3	739.8	35,031	174.5	26	45.7	824.8	28,938	157.6

**GX2D**	**115**	7	2009	4	25.4	339.7	6,169	123.6	16	38.2	709.6	17,104	132.8

**MH2D**	**171**	35	2008	4	32.7	1,255.6	32,564	127.8	23	44.0	822.0	24,558	150.9

**DR2D**	**239**	13	2008	4	19.0	219.0	26,084	93.7	23	42.0	695.5	28,769	145.5

**DR3D**	**265**	54	2008	4	43.2	883.1	55,819	217.8	29	44.6	737.9	32,592	154.5

**CH2M**	**269**	183	2008	20	43.1	1,404.7	35,222	156.2	30	48.5	839.4	34,417	169.5

**CH2M**	**270**	138	2008	16	47.5	1,646.9	28,088	170.3	30	48.3	842.1	34,136	168.2

**CH2M**	**271**	131	2008	16	40.0	1,745.1	22,248	140.2	29	46.7	808.5	32,671	163.1

The estimates of stand level TPH, Board Feet (BF)/ha, and Metric Tons of Carbon (MgC)/ha showed no statistically significant difference between the past stand delineation estimate and aggregating the current stratification system to the old stand boundaries except for basal area (paired t-test p-values: BA = 0.034, TPH = 0.23, BF/ha = 0.81, metric tons Carbon/ha = 0.7).

## Discussion

### Selection of Grid Size

The first step in partitioning the variability of the GRF was to establish a grid across the whole property. Many LiDAR driven forest inventories in past studies have used stem-mapped plots to correlate ground data with remote sensing data by using the actual location of trees and their crowns to build models that relate to the remotely sensed crown polygons and crown heights [37]. In this application however, variable radius plots were used to correlate the vegetation and the cell variability recognized by the LiDAR imagery. Stem mapping was not chosen because it would have been prohibitively expensive due to the high number of stems per ha and the steep terrain. However, because variable radius plots were used it is difficult to know the optimal size for grid-cells given that the size of the plots is variable [65].

The exercise of choosing the size of the grid cells is dependent on several factors. The first consideration is the ability to accurately locate sample plots using handheld GPS units. The GPS units used by the inventory cruisers have accuracies that exceed 10 m (33 feet) 95% of the time [71]. The second factor when choosing the grid size is finding the optimal cell size to reduce the variability between the remote sensing data and the measured plot data. Past studies have shown that it is important to choose a grid size that best matches the size of the plots installed [41,48]. van Aardt et al. [64] also explored this question using an object based approach (as opposed to pixels, objects are non-uniform areas of similar characteristics) and found only a small loss of accuracy with increasing object size. Pesonen et al. [72] have also examined the optimal fixed area grid cell size but for that study focused on finding the optimum grid cell size when estimating coarse woody debris as opposed to standing trees.

Approaching the question of the optimum size to best relate plot data to remote sensing data, Gobokken and Næsset[63] used a Monte Carlo analysis to explore the optimal size of fixed area plots in developing accurate forest inventory estimates. This analysis is similar to our current question but may be difficult to implement in practice as the plots may already be measured or it may not be appropriate to change the plot design mid-sample.

In this case, a 4.6 m^2^/ha (20 ft^2^/acre) basal area factor (BAF) prism was used on each plot. Generally, a 4.6 BAF prism samples about 0.04 ha but this will change depending on the size of the trees. To test this, the average of the limiting distances of each tree measured in all of the variable radius plots was calculated and the median plot size based on this analysis was determined to be 0.036 ha. However, larger trees would likely be outside of grid cells that are 0.4 ha or smaller. In addition, there is a greater chance that the location of the plot in the field would fall outside of the target grid cell due to the variability in the estimates of location made by the handheld GPS units. Therefore, grid cells less than 0.4 ha (1/10th acre) were deemed too small.

As the grid cell size increases to sizes larger than 0.4 ha, the variability of the forest within the cell (and hence the remote sensing data) increases. Because of this, it was hypothesized that any model that relates plot metrics to summarized grid cell remote sensing data will theoretically perform worse as the size of the cell increases to sizes larger than the plot. For these reasons, a 0.04 ha cell size was used as it was deemed to be the smallest cell size that would contain a 4.6 BAF plot and the location error associated with the handheld GPS units, and result in minimal within cell variability.

**Figure 5:**
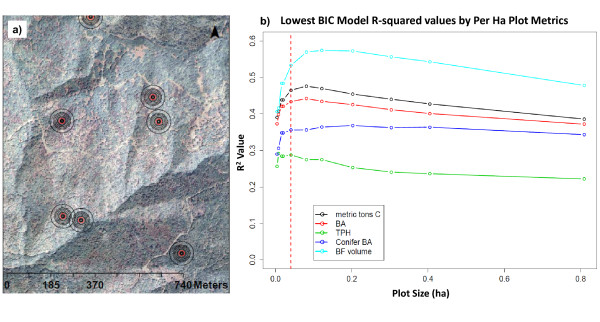
**Optimum Grid Cell Size Results**. a) Remote sensing sample units of different size. Red circle represents 0.04 ha. b) Results of lowest BIC model selection approach using an exhaustive search of all potential model permutations. Dashed red line shows 0.04 ha size.

After further analysis following the completion of the inventory, the 0.04 ha grid cell size may have been slightly too small to create the strongest relationship between plot values (e.g. - BA, TPH, volume, carbon, etc), topographical data (elevation, slope, aspect), and remotely sensed data (e.g. - orthophoto band intensity). The optimal grid cell analysis was undertaken after the inventory was completed as a means to assess if the pixel size used was the best size and to inform future projects. The approach outlined below is one method that could be used to decide on the size of pixels to divide a forested area into and would ideally be used prior to the final sample. To determine the optimal grid cell size, a sample of the remotely sensed data was taken at each field plot point with a series of increasing circular areas (see Figure 5a). The mean and standard deviation of all remotely sensed variables for each circular region for each data set was then calculated for each size circle. Once the remote sensing derived data had been summarized to each sample size, an exhaustive model selection routine was run to find the best model assuming the best model was defined using Bayes Information Criteria (BIC) [73,74]. The BIC was used as the metric of model performance because it does not assume that a relationship between explanatory and predictor values exists and has a larger penalty with larger data sets [75]. Once the model with the lowest BIC was chosen for each circular area the amount of variation explained was graphed relative to each other sample size (Figure 5b). In this way, an objective approach to model selection can provide a metric to judge which size grid-cell is optimal. Based on the results seen here, it seems the optimal cell size was about 0.08 ha (1/5 acre). This would be slightly larger than the cell size actually used.

### Sampling Intensity by Strata and Plot Location within Strata

The optimal sampling intensity of the final sample can be determined using a Neyman allocation of plots (or an optimal allocation of plots if the plots have variable costs in different strata) using the traditional approach to estimating the appropriate sample size [66]. For strata that do not have an adequate initial sample to have confidence in the estimate of the sampling variability, an estimate of the variability of the strata can be found using the remote sensing data for that strata compared to the other strata. In this case, using the models developed from the initial strata to populate the cells of the under-sampled strata an estimate of the population variance can be found and used to calculate the optimum sample size. Plots are then randomly located within the strata.

### Future Directions

#### Management Planning

Using this new approach will be a significant departure from how forest planning traditionally proceeds using a stand based approach. Using a grid-based stratification, analysis of given forest areas in these small units can provide more fine-grained information about any given area. For example, when laying out timber harvest plan boundaries, these forest strata can be used to more accurately understand current stocking and forest conditions and allow for better layout of plan boundaries and a better description of pre-harvest conditions and habitat.

Although this stratification approach provides much higher resolution data in terms of understanding current forest conditions, there are several challenges to using this approach. To begin, this grid system does not lend itself to easy modeling of future management because the stand structure (400 m^2 ^pixels) are not logical management units. Secondly, although we have more confidence in the total volume of any given cell across the property, there may be more variation in the species composition within a strata type. This is a result of the fact that total volume, not merchantable volume, was the variable whose variation was optimized during the creation of strata. In future efforts, both total volume and merchantable volume should be considered when creating strata boundaries.

### Sampling of Harvest or Disturbance

As mentioned above, this strata system provides a highly flexible and accurate picture of current forest conditions. Moving forward, as areas are harvested or undergo natural disturbance however, sampling will revert back to a more traditional harvest area (stand) based approach. The reason for this is twofold. First, the cost of collecting new remote sensing data annually prevents the collection of the necessary data to drive this stratification process. Second, the known THP boundaries or disturbance events can be used to generate more accurate stand boundaries. Therefore, future sampling will proceed by first delineating the disturbed area and then sampling within this area to estimate the standing forest stocks post disturbance.

### Ecological Monitoring

We anticipate that the canopy height model will be used in the future to generate a revised Northern Spotted Owl (NSO) habitat model to assist in management of the NSO. One of the benefits of this small grid system is that the final plot data can also be used to develop full parametric models for any variable of interest. In some cases (e.g. canopy cover), models are not required as the variable in question is measured directly by the LiDAR data. In this case, the canopy cover found in trees greater than 28 cm (11in) DBH will be modeled to inform the classification of NSO habitat [76] (traditionally this classification was based on lower resolution ocular estimates).

### Pre-Aggregation for Process Modeling

Hawbaker et al. [62] show that there is a need for ALS to be leveraged across larger landscapes and that ALS can help to create more accurate estimates of biophysical variables at a landscape scale by helping to better define the sampling design used. The method of sampling and stratification outlined in the following section can also be used to both validate process models and to serve as a pre-aggregation framework across a large landscape. Although this method uses ALS and optical remote sensing data with continuous coverage across the landscape it could also be applied to larger scales using a variety of data sources with or without full coverage. Specifically, by running models based on a small set of strata instead of in each grid-cell across a region much more efficient and rapid estimates of ecosystem state can be generated.

Lefsky et al. [32] have shown the value of using ALS combined with Landsat data to construct independent estimates of landscape net primary productivity and net ecosystem productivity to compare with light-use efficiency models or biogeochemistry models. Their work used remote sensing data collected over time to detect change. The strata system developed here will serve as the basis for future biogeochemistry model runs that will also attempt to better estimate ecosystem carbon fluxes at the GRF.

## Conclusions

The method described below not only provides a cost effective and flexible approach to stratifying a forest but also has been designed and applied in the context of the requirements of existing forest carbon project protocols. This is highly valuable given that monitoring, reporting, and verifying carbon stocks and fluxes at a project level is the single largest external cost of a forest carbon offset project. Although currently the use of LiDAR approaches for smaller scales still is not cost effective, using a method like this one at scales larger than 10,000 ha (25,000 acres) may pay for themselves by reducing the cost of the field inventory required.

Additionally, the use of both parametric approaches (to develop models from the initial sample) and non-parametric approaches (to partition the variables of interest into strata) provides more power to determine the optimum sampling intensity and location across a large ownership. Furthermore, the 2 stage sample allows for the optimum grid cell size to be found.

For management decisions, this ALS and optical remote sensing stratification design and high-resolution grid allows for more accurate estimates of volume at any scale larger than a 0.04 ha grid cell (1/10 acre). This new strata layer and the data associated with it will serve as a baseline of forest conditions against which future management at the Garcia River Forest can be compared and assessed. Additionally, because of the flexibility built into this method, it can be scaled to much larger or smaller spatial extents. This is valuable for planning both local and larger scale ongoing management and monitoring activities.

## Methods

### Study Site

The Garcia River Forest (GRF) project is a 9,623 ha (23,780 acre) forest located in Mendocino County, California northwest of the town of Boonville. This forest is owned by The Conservation Fund (TCF) and is protected by a conservation easement held by the Nature Conservancy (TNC). The goals of the project are to conserve and restore highly productive and biologically diverse forests and streams, and to implement sustainable forest management practices that support the local economy [77]. This region is historically dominated by a mix of redwood (Sequoia sempivirens)and Douglas-fir (Pseudotsuga menziesii) trees but due to decades of industrial timber management and intensive harvesting of this forest there is now a higher than natural amount of Tanoak (Lithocarpus densiflorus) in traditionally conifer dominated stands.

Due to the past management of the GRF, most stands have a mix of young 2nd or 3rd growth redwood and Douglas-fir trees with high proportions of tanoak. Most areas are heterogeneous within stand boundaries and these conditions are the norm across the full ownership. Past management consisted mostly of "thinning from above" - removing the larger, better trees from most stands - and as a result most stands are made up of small, young trees.

Because of the state of the forest today, it is difficult to use a traditional stand mapping approach to delineate areas that are substantially similar. The result of applying the traditional air photo interpretation approach to stand mapping in this forest resulted in the creation of large stands that have high degrees of within stand variability and don't always relate to logical management units (see Figure 2).

### Field Data

#### 2009 Data (used for comparison to 2010 stratification results)

The existing inventory consisted of plots installed over several years using several different cruising protocols. Both variable radius plots and fixed area plots were installed across the property from 1999 to 2009. Most recently (2006 to 2008), all cruising occurred on a 400 by 400 meter (20 by 20 chain) grid that covered the full ownership using 4.6 Basal Area Factor prisms (Table 2). The complete inventory from 1999 to 2009 was grown forward to 2009 using the Forest Projection and Planning System growth and yield model to compare property level estimates in 2009 to the new stratification method in 2010. However, only plot data from 2008 and 2009 was used to compare individual stand level estimates to aggregated pixel estimates (see table 5).

The old stand layer was a traditional timber stand typing done by head's up digitizing stand boundaries using color imagery (acquired in 2004) of the forest. Each stand was then placed within a strata that described the dominant tree size and species based on the professional judgment of the land manager. The old strata types had 3 fields: a 2 digit species code that described the dominant species or species mix, a 1 digit size-class code that described the dominant tree size, and a 1 digit canopy density code that described the degree of canopy closure.

#### 2010 Data (used for stratification)

The 2010 inventory data was collected between June and September of 2010. It consists of 810 variable radius plots that use a 4.6 m^2^/ha (20 ft^2^/acre) basal area factor (BAF) prism to measure trees at least 14 cm (5.5 inches) DBH. All plots have height measured on all trees (both live and dead) that are tallied in the variable radius plot. In addition to the trees measured in the prism plot, there is a 0.04 ha (1/10th acre) circular plot for understory vegetation, a 0.004 ha (1/100th acre) plot to measure regeneration (trees less than 14 cm DBH), and a 30.5 m (100 ft) transect to measure down woody debris. Table 4 summarizes the current inventory data and the past inventory data. The past 2009 inventory and stand layer was used as a baseline against which to compare the new 2010 ALS based stratification and inventory system.

The field sampled plots for the preliminary sample (199 plots) were a random selection of a 400 m by 400 m (20 by 20 chain) grid. Table 6 lists the summary statistics for the preliminary sample.

**Table 6:** Initial 199 Plot Summary Statistics

Variable	Min	Mean	Max
**BA (m**^**2**^**/ha)**	0	40.73	116.1

**TPH (Trees Per ha)**	2	2,339	14,944

**% conifer BA**	0	56.6	100

**Average height (m)**	7	29	62

### Remote Sensing Data

Both color-infrared imagery and LiDAR data were collected for the full property (Table 7). The color-infrared imagery has 0.6 meter (2 foot) resolution with horizontal accuracy less than 1 meter. The raw LiDAR returns range from 2.5 to 27 returns per square meter with at least 5 returns per square meter for forested areas. The LiDAR data exceeds 15 cm of vertical accuracy and 50 cm of horizontal accuracy. The LiDAR returns were summarized to make a 1 square meter digital elevation map and a 0.5 square meter canopy height model. The CHM is gridded to 0.5 m^2 ^and based on the interpolated "highest" return within each pixel. In addition to these grids, the LiDAR data were used to generated a crown polygon layer for the full GRF. The crown polygon layer was created using a watershed transformation algorithm applied to the CHM that segmented individual tree crowns that are isolated in height from adjacent regions.

**Table 7:** Summary of Remote Sensing Data Collected in 2009

	Color Infrared	Light Detection And Ranging
**Acronym**	**CIR**	**LiDAR**

**Date Collected**	7/1/2009	

**Source**	Fixed-wing aircraft	

**Instrument**	Digital Mapping Camera from Zeiss/Intergraph Imaging	ALTM Gemini from Optech Incorporated

**Scale**	Full ownership	

**Projection**	North American Datum 1983 UTM zone 10N	

**Resolution**	0.6 meter	5 returns/square meter, 24° field of view, 0.44 postings/square meter.

**Spectrum**	visible and near-infrared (380 nm to 2500 nm)	near-infrared (760 nm to 2500 nm)

**Accuracy**	Horizontal accuracy sub 1 meter	Horizontal accuracy sub 50 cm Vertical accuracy sub 15 cm

**Data Form**	4 bands: red, blue, green, and near-infrared	Discrete Waveform with classified returns (ground, mid-canopy, upper-canopy)

**Products**	Ortho-rectified 4 band CIR	All and first return LiDAR (raw data) 1 m^2 ^Digital Elevation Model (DEM) 0.5 m^2 ^Canopy Height Model (CHM)Crown Polygon Layer

### Description of the Method

#### Data Summarization to 400 m^2 ^pixels

The first step before any analysis, inventory, or stratification could occur was to summarize all of the remote sensing data to the 400 m^2 ^grid cells. This involved finding the average and variance of all of the remote sensing data sets (e.g. CIR, RGB, canopy height, crown polygons, topography variables - slope, aspect, elevation, and a whole suite of other variables derived from the remote sensing data in both the spatial and frequency domains). The complete set of variables used for the analysis and a brief description of them are listed in the appendix.

The source data for the cell summaries used in the stratification come from two passive image datasets and summarized LiDAR. The three image sets (CIR, RGB and CHM) were processed with MATLAB's image processing toolbox [78]. The image processing routines work in two domains; the spatial, and the frequency [79]. The pixels from the image data sets are about 0.6 meters on a side. The CHM is treated as a gray scale image where height above the ground is scaled to the gray scale.

### Initial Plot Installation

To develop the final stratification, a set of "training" field plots were installed to find the relationships between plot data and the cell data (e.g. volume, carbon, basal area). To do this, an initial set of 199 plots were installed across the GRF. A random sample of points located at the intersections of a 400 m by 400 m (20 by 20 chain) grid was chosen to cover a broad spatial area.

### Variable Reduction using Principle Component Analysis (PCA)

The 400 m^2 ^cell data was summarized using principle component analysis to reduce the number of variables. Factor analysis was used to determine how many of the principle components should be retained [80]. Table 8 lists the amount of variation explained by the first eight and the next eight principal components in the each of the image datasets. Based upon the reduction in explained variance and the need to keep the preliminary sample small, the first eight component vectors were selected to represent the data sets in the preliminary sample.

**Table 8:** Principle Component Decomposition of the Imagery Datasets

Image set	Variance explained by first eight	Variance explained next eight
**RGB**	76.00%	13.40%

**CIR**	75.10%	13.70%

**CHM**	72.60%	14.70%

The original optical data consisted of 4 bands of data: blue, red, green, and NIR reflectance values. Although it would be possible to analyze this data by combining all 4 bands into one image, instead this optical data was used to create two images: a color-infrared (CIR) image and a Red-Green-Blue (RGB) image. The CIR image combines the red, green, and NIR values. There are two reasons why the red and green bands were included in both the CIR and RGB datasets: 1) to check that the atmospheric correction was applied correctly and 2) to have finer control of the linear combination of the data when conducting the analysis.

Since two of the color bands (red and green) are present in both the CIR and RGB image data, a correlation analysis was conducted to determine the amount of overlap between the principal components of the two datasets. The Pearson correlations with p-values less than 0.05 have an asterisk in Table 9.

**Table 9:** Correlation Analysis between the CIR and RGB Principle Component Datasets

PrinComp	RGB1	RGB2	RGB3	RGB4	RGB5	RGB6	RGB7	RGB8
**CIR1**	0.925*	-0.128	-0.418*	0.137	-0.144*	-0.282*	-0.011	-0.08

**CIR2**	-0.072	0.977*	0.091	-0.067	0.064	-0.237*	-0.034	-0.048

**CIR3**	-0.09	-0.243*	0.158*	0.736*	-0.1	0.029	-0.252*	0.068

**CIR4**	-0.273*	0.068	0.891*	-0.185*	-0.189*	0.382*	-0.328*	-0.018

**CIR5**	-0.249*	0.101	0.371*	-0.254*	0.931*	0.123*	-0.11	0.0002

**CIR6**	-0.226*	-0.201*	-0.079	-0.237*	-0.206*	0.840*	-0.056	0.087

**CIR7**	0.052*	-0.019	-0.378*	-0.041	-0.009	-0.319*	0.890*	-0.084

**CIR8**	-0.170*	-0.008	0.066	-0.051	0.06	-0.177*	0.337*	0.880*

A quick scan of Table 9 shows that, as expected, some of the principal components are highly correlated. This correlation reduces the efficiency of variable screening methods applied to this data, meaning that more plots will be required to achieve the same level of certainty. The impact of the correlations was examined by repeating the parameterization of the models described below with both data sets separately and then both together.

### Parameterization of Models to Relate Remote Sensing Data to Initial Inventory

The data collected in the first 199 plots was then correlated to the reduced set of remotely sensed variables found using the PCA. Several models were built that related remotely sensed data to the measured plot data in each sampled 400 m^2 ^cell. However, only the BA regression model, multiplied by each cell's average canopy height, was used by the Tabu Search Algorithm to develop the initial strata. The BA model was then used to predict the BA in all of the 240,410 400 m^2 ^cells across the full ownership. The .5 m^2 ^resolution Canopy Height Model (CHM) was averaged across each 20 by 20 m pixel and used to estimate the average canopy height in each pixel (no model was required as this is directly measured by the LiDAR data).

Stepwise procedures have been found to produce poor variable screens [81]. This is partially due to the repeated comparisons not representing the proper elimination probabilities [82]. However there are other problems with the method such as the parameter estimates being biased high, and the standard error of the estimates being too low. This results in F and chi-squared statistics not having the desired distributions [83]. Based upon this the Lasso method [84] was used for the variable screening of the predictive models. The Lasso is a penalized least squares method which selects a set of regression coefficients (β^*Lasso*^) as the coefficients that minimize the following equation:

$${\hat{\beta }}^{Lasso}=argmin_{\beta }\left\{\overset{n}{\underset{i=1}{\sum }}{\left(y_{i}-\beta_{0}-\overset{p}{\underset{j=1}{\sum }}x_{ij}\beta_{j}\right)}^{2}+\lambda \overset{p}{\underset{i=1}{\sum }}|\beta_{i}|\right\}$$

In the above equation, y is an n-length vector of the response variables; X is an n by p matrix of predictor variables. β_0 _and β_j _are the standard regression intercept and coefficient vectors while the last term is a penalty term applied to each coefficient - lambda is the penalty multiplier that is applied to each estimated coefficient.

To ensure that no single predictor swamps the effects of others, the matrix of predictors (X) is centered and scaled, and then λ is chosen by cross-validation. This means that a portion of the plots are held back from the regression and these plots are then predicted by the resulting regression. The Lambda value is iteratively adjusted to produce the lowest prediction error of this cross-validation. The Lasso serves as a variable selection methodology by selecting few predictors thus alleviating problems attendant to having many potential predictors compared to the number of observations. Furthermore, since the Lasso tends to select only a few of a set of correlated predictors, it also helps reduce problems with spatial correlation [84].

### Final Stratification Using Supervised Classification

Based on the predictions of the BA model described above, an optimal binning process [85,86] was used to create bins (strata) for each cell based on the product of A and height. The stratum for each cell was determined by minimizing the amount of variation of the product of BA and height in each strata. The product of BA and height is highly correlated to volume and therefore cells within a given strata have similar volume totals. This classification method is considered supervised since it is driven by the initial inventory data collected across the GRF.

Once the supervised classification was completed, to prevent any strata from being less than 4.05 ha (10 acres) in size, an algorithm was applied to swap grid cells that were on the "edge" of each strata into neighboring strata (considering the nearness according to BA, height, Trees Per Hectare (TPH), and % conifer BA). The goal of this algorithm was to minimize the variation covered within a given strata while reducing the total number of strata.

### Selection of Remaining 611 Sample Plots Based on Final Stratification

The final 611 plots were randomly placed within each final stratum in proportion to the variability in product of BA and height. This sampling design is a classic post-stratification design and therefore uses stratified random sampling estimators [66,87].

## Abbreviations

ALS: Airborne Laser Scanning; BA: Basal Area; BF: Board Foot Volume using the USFS board foot volume calculations [88]; CHM: Canopy Height Model; CIR: Color Infrared optical data; GRF: Garcia River Forest; ha: hectare = 10,000 m^2^; LiDAR: Light Detection and Ranging; PCA: Principle Component Analysis; RGB: Red, Green, and Blue optical data; THP: Timber Harvest Plan; TNC: The Nature Conservancy; TCF: The Conservation Fund; TPH: Trees Per Hectare

## Competing interests

'The authors declare that they have no competing interests.

## Authors' contributions

JG: final write-up, conceptual development, analysis of results, description of future uses, development of optimal grid-cell size analysis. MH: processing of raw data, development of method and application to data, write up of method section, critical review and analysis of results. JC: conceptual development, review, description of future uses.

All authors read and approved the final manuscript.

## Appendix - Variables Used

### Topographic Variables

1. Average elevation

2. Variance of the elevation of the cell.

3. Average aspect

4. Variance of the aspect of the cell.

5. Average slope

6. Variance of the slope of the cell.

7. A measure of the difference between the actual topography of the cell and a plane joining its corners.

### Crown Segment Variables

1. Number of polygon centroids within a cell (pcount).

2. Average of the maximum height above the ground for the polygons (cell height).

3. Variance of the maximum height above ground for the polygons.

4. Crown closure as the percentage of the cell area covered by polygons.

5. Curvature of the cell in relation to the eight nearest neighbor cells (NLN).

6. Average LiDAR first return intensity for the cell.

7. Variance of the LiDAR first return intensity for the cell.

8. Average intensity of the infrared band of the CIR data fused to the polygons.

9. Variance of the intensity of the infrared band of the CIR data fused to the polygons.

10. Average intensity of the red band of the RGB data fused to the polygons.

11. Variance of the intensity of the red band of the RGB data fused to the polygons.

12. Average intensity of the green band of the RGB data fused to the polygons.

13. Variance of the intensity of the green band of the RGB data fused to the polygons.

14. Average intensity of the blue band of the RGB data fused to the polygons.

15. Variance of the intensity of the blue band of the RGB data fused to the polygons.

16. Ratio of the infra-red to red bands.

17. Normalized difference vegetation index(NDVI = (IR - red)/(IR + red)).

### Image Variables

Image set variables consist of two types of analysis; spatial and frequency. Spatial analysis quantified the relationships between the pixels based upon their location with respect to one another. Frequency analysis characterizes the spectral characteristics of the pixels both in relation to one another and to standard frequency distributions.

There are no known relationships between these summary variables and the structural characteristics of the vegetation from which the light was reflected. This is an intriguing line of research but time has not yet been allotted for its pursuit. The CHM was treated as a greyscale image for this analysis.

### Spatial Domain

1. Image profile analysis consisting of summaries of the eight vectors originating at the center of the image and radiating to each corner and the middle of each edge. This includes the mean, variance, median, skewness, kurtosis, entropy, mean absolute deviation, median absolute deviation of the pixels on the profile.

2. Image pixel analysis, the pixel based mean, variance, median, entropy, mean and median absolute deviation from a unit vector.

3. Histogram analysis of the image.

4. Sum of the Hough lines within the image. This has been used to identify plantations, and roads.

5. K-mean clustering of the color bands in the image.

6. The ratio of the number of pixels in two color groups is compared using a quadrant analysis.

7. Number of cluster centers arising from the first group from the quadrant analysis.

8. The fraction of shadow.

9. The values of a three parameter Weibul fit to the image intensity histogram. The number of local maximum points and the location of the first three local maximums in a three dimensional histogram constructed in l, a, b color space.

10. The correlation, contrast, busyness, and texture strength of a neighborhood grey level difference matrix.

11. Neighborhood occurrence test based on eight offsets and compared with the Spectral Information Divergence.

12. Contiguous region analysis including the average area, eccentricity, extent, orientation, and solidity of two size classes of blobs.

### Frequency Domain

1. The ratio of the geometric mean to the arithmetic mean of the frequency space image.

2. Comparison of a vector of texture based properties such as contrast homogeneity correlation and energy using the gray scale co-occurrence matrix for a fixed diagonal offset on an image to a spectral information divergence.

3. Comparison of a vector of texture based properties such as contrast homogeneity correlation and energy using the gray scale co-occurrence matrix for a fixed diagonal offset on an image to a spectral angle measure.

### Reduced variable set

1. CIR1-CIR8 the first eight principle components of the color infrared image

2. RGB1-RBG8 the first eight principle components of the true color image

3. LI1-LI8 the first eight principle components of the canopy height image
